# Malaria and Money

**Published:** 1917-01

**Authors:** 


### Malaria and Money.

The States in which malaria prevails are beginning to figure how much money they are losing from malaria. The farmer who is sick can't work, and, if he can't work, he can't make the money with which to take care of himself and family. Instead of being a producer he becomes a burden on some one or on the State. The loss from sickness due to malaria is said by actual computation to cost only about half of the loss due from inefficiency. Malaria prevails most in the swampy sections of the South, where lands are subject to overflow, are undrained, and are more adapted to breeding disease-carrying mosquitoes than to rearing healthy children. These are sections which are avoided as residence places by all people except the very poor, and the poor become a public burden more quickly than would those who have means with which to tide over temporary reverses caused by sickness. They are the sections in which however much practice physicians may get, the remuneration for it is very small.

It is estimated roughly that the loss in the United States every year due to malaria is more than $100,000,000. This is the money loss, and permanent inefficiency caused by the disease is not included in the estimate. Every Southern State is a great economic loser. It is a kind of loss from which nobodyy gains anything; certainly physicians do not profit by a practice of that kind even if they should be so mercenary as to be indifferent to remedying an evil from which they might profit, and they will willingly and gladly co-operate with any State movement to eradicate malaria.

That something should be done wherever it prevails is admitted; the poor people in those sections are unable to help themselves. Even the local governments are too poor generally to undertake so great a work. The only hope is that State governments may come to the relief of the malaria-infected sections. If it is not done, they will continue to produce less and less, those who can leave will abandon them, and malarial districts will become a permanent economic liability instead of being an asset.

